# Racial differences in treatment and outcomes in multiple myeloma: a multiple myeloma research foundation analysis

**DOI:** 10.1038/s41408-020-00347-6

**Published:** 2020-08-07

**Authors:** Benjamin A. Derman, Jagoda Jasielec, Spencer S. Langerman, Wei Zhang, Andrzej J. Jakubowiak, Brian C.-H. Chiu

**Affiliations:** 1 Section of Hematology/Oncology, University of Chicago Medical Center, Chicago, IL USA; 2 Department of Public Health Sciences, University of Chicago, Chicago, IL USA; 3 Department of Preventive Medicine, Northwestern University, Chicago, IL USA

**Keywords:** Cancer epidemiology, Myeloma

## Abstract

Findings on racial differences in survival in multiple myeloma (MM) have been inconclusive. We assessed differences in outcomes between White and Black individuals among 639 newly diagnosed MM patients in the MM Research Foundation CoMMpass registry with baseline cytogenetic data. Survival curves were constructed using the Kaplan–Meier method. Hazard ratios and 95% confidence intervals were derived from Cox proportional hazard regression models. Age, gender, and stage were similar between Whites (*n* = 526) and Blacks (*n* = 113). Blacks had inferior overall survival (OS) compared with Whites and were less likely to receive triplet therapies or frontline autologous stem cell transplant (ASCT). The following factors were significantly associated with inferior OS in multivariate analysis: higher international staging system (ISS) score, ≥1 or ≥2 high-risk cytogenetic abnormalities (HRCA), high-risk gene expression profile (GEP), and lack of ASCT. Multivariate analysis in the Black subset found that only lack of ASCT was significantly associated with inferior OS. The receipt of both triplet induction and ASCT only partly abrogated the effect of race on survival. HRCA did not track with survival in Blacks, emphasizing the need for race-specific risk prognostication schema to guide optimal MM therapy.

## Introduction

Multiple myeloma (MM) is part of a spectrum of monoclonal plasma cell disorders with an age-adjusted incidence of 7.0/100,000 in the United States and comprising 1.8% of all new cancer diagnoses in 2020^1^. Unlike the well-recognized two-to-threefold higher incidence rate of MM among Black individuals compared with Whites^2–6^, findings on racial differences in mortality and treatment outcomes have been inconclusive. Population-based studies using surveillance, epidemiology, and end results (SEER) registry and studies using trial data have suggested either similar or superior relative survival for Blacks compared to Whites with MM^2,7–11^.

These findings are surprising in light of the fact that Blacks face barriers that may lead to inferior survival, including lower socioeconomic status and lower likelihood to receive contemporary MM agents or undergo autologous stem cell transplant (ASCT)^9,12^. In a retrospective analysis of 15,717 patients with MM in the Veterans Association (VA) health care system with equal access to care between 2000 and 2017, Fillmore et al.^13^ found that Blacks had better overall survival (OS) compared with Whites, even after adjusting for age, sex, rurality, income, stage, transplantation, and induction therapies. A similar superior survival in Black individuals with MM after ASCT was also reported by Sweiss et al.^14^. In contrast, several studies have shown similar OS between Blacks and Whites, though this is despite later access to novel therapies or ASCT^9,15–18^.

One potential explanation for the racial differences in outcomes may lie in the distribution and impact of cytogenetic or molecular mutations that have prognostic significance. One multi-institutional study reported that the cytogenetic abnormalities *t*(11;14), *t*(4;14), monosomy 13, and monosomy 17 were less common in Blacks^19^. Analysis of the Multiple Myeloma Research Foundation (MMRF) CoMMpass data set found that Blacks had a higher frequency of *BCL7A, BRWD3*, and *AUTS2* mutations, and a lower frequency of *TP53* and *IRF4* mutations compared with Whites^16^. Despite examining these differences, no study has holistically evaluated the racial differences in outcomes according to the complex interplay of prognostic indices, cytogenetics, and modern treatment approaches. To address this quandary, we investigated outcomes between Blacks and Whites in a cohort of 639 MM patients receiving modern treatment approaches.

## Materials and methods

### Study population

We obtained the data on newly diagnosed MM patients from the Multiple Myeloma Research Foundation (MMRF) CoMMpass registry (NCT01454297, version IA13). The CoMMpass study was initiated in 2011 as a large-scale prospective observational study in MM that has collected tissue samples, genetic information, quality of life, and clinical outcomes from over 1100 patients with newly diagnosed MM at 90 different sites worldwide. Each patient is followed every 6 months for a total of 8 years. Bone marrow samples were collected at enrollment, during response to therapy, and at relapse.

From an initial 1,154 patients with accessible data in the CoMMpass registry, 515 were excluded due to incomplete cytogenetic data (*n* = 274), missing demographic data (*n* = 172), or self-identified race other than Black or White (*n* = 69). This resulted in a total of 639 evaluable patients that made up the study population. Fifty patients reported being of Hispanic/Latino ethnicity, all of whom reported to be of White race and all of whom had >60% European ancestry according to the calculated ancestries by Manojlovic et al.^16^. These patients were included in the current report given that their exclusion did not materially change point estimates and overall findings.

### Cytogenetics and treatment data

The CoMMPass registry inferred cytogenetic changes from the next-generation sequencing (NGS) data; a deletion required that 21% of cells have at least a one copy deletion, a gain required that 23% of the cells have a one copy gain, and translocations required at least 30% of cells having the event. Abstracted data included pre-treatment demographics, International Staging System (ISS), baseline MM parameters, cytogenetics, induction regimen, autologous stem cell transplant (ASCT) and maintenance therapy use, progression-free survival (PFS), and OS.

Race was determined based on self-reported race. High-risk cytogenetic abnormalities (HRCA) were defined according to the International Myeloma Working Group classification as any of the following: deletion 17p/*TP53*, 1q gain or amplification, *t*(4;14), *t*(14;16), and *t*(14;20)^20^. High risk by UAMS70 gene expression profiling from the CoMMpass data set was determined using an independent cutoff in a manner similar to what was previously done^21^.

### Statistical analysis

Chi-square or Fisher’s exact test were used for comparisons of categorical variables and the *t* test for continuous variables. We defined PFS as the time from diagnosis until progression or death. OS was defined as the time from diagnosis until death from any cause. Survival curves were constructed using the Kaplan–Meier method and compared with the log-rank test. Cox proportional hazard models were computed to estimate hazard ratio (HR) and 95% confidence interval (CI) for association between pre-treatment variables and outcomes. Age was evaluated as both a continuous and categorical variable for age-adjusted Cox analysis and the methods generated similar findings; therefore, age was treated as a categorical variable for the multivariate analysis. Multivariate analysis was performed using all variables that were significantly associated (*P* < 0.05) with PFS and OS by univariate analysis within each group, in addition to HRCA and high risk by UAMS70 given clinical interest in and biologic plausibility of these variables. Data analysis was carried out in Stata V15.0 (StataCorp).

## Results

A total of 639 MM patients (113 Blacks and 526 Whites) were identified in the MMRF CoMMpass registry with complete baseline cytogenetic data available. Median age was 65 years for Whites, and 63 years for Blacks (*P* = 0.2); 319/426 (61%) Whites and 69/113 (61%) Blacks were male (*P* = 0.9). There was a similar distribution of HRCA and the number of HRCA between Blacks and Whites (Table 1). There were also no between-race differences in ECOG performance status, ISS/Revised-ISS stage, or bone marrow monotypic plasmacytosis percentage.

**Table 1:** Characteristics of MMRF patients.

	White (*n* = 526)	Black (*n* = 113)	*P*-value
Age, median (range)	65 (38–89)	63 (34–87)	0.2
Male gender	319 (61%)	69 (61%)	0.9
*No. of high-risk cytogenetic abnormalities*			0.6
0	254 (48%)	58 (51%)	0.6
1	200 (38%)	43 (38%)	0.5
2+	72 (14%)	12 (11%)	0.4
*High-risk abnormalities*			
* t*(4;14)	64 (12%)	13 (12%)	0.8
* t*(14;16)	21 (4%)	6 (5%)	0.5
* t*(14;20)	7 (1%)	3 (3%)	0.3
Deletion 17p	67 (13%)	11 (10%)	0.4
1q gain	193 (37%)	34 (30%)	0.2
*ISS, n*	505	109	0.2
1	179 (35%)	30 (28%)	
2	166 (33%)	43 (39%)	
3	160 (32%)	36 (33%)	
*ECOG performance status*			0.8
0	164 (34.5%)	34 (33.7%)	
1	231 (48.5%)	45 (44.6%)	
2	56 (11.8%)	15 (14.9%)	
3	21 (4.4%)	6 (5.9%)	
4	4 (0.8%)	1 (1.0%)	
*Estimated GFR (mL/min/1.73m*^*2*^*), median (IQR)*	69 (47–86)	65 (42–91)	0.17
eGFR <60 (%)	205 (39%)	48 (42%)	
eGFR 60–90 (%)	216 (41%)	36 (32%)	
eGFR >90 (%)	103 (20%)	29 (26%)	
N/A	2 (<1%)	0 (0%)	
*Induction therapy*			0.001
Any triplet	384 (73%)	62 (55%)	<0.001
PI+IMiD triplet	240 (46%)	40 (35%)	0.05
Alkylator-based triplet	144 (27%)	22 (20%)	0.1
Doublet	118 (22%)	46 (41%)	<0.001
Other	24 (5%)	5 (4%)	1
*Best response to induction therapy, n*	512	109	0.2
<VGPR	290 (57%)	69 (63%)	
≥VGPR	222 (43%)	40 (37%)	
Received triplet + ASCT	231 (44%)	37 (33%)	0.04
Received firstline ASCT	260 (49%)	44 (39%)	0.04
+Post-ASCT maintenance	157 (60%)	26 (59%)	0.9

*ASCT* autologous stem cell transplant, *ECOG* Eastern Cooperative Oncology Group, *GFR* glomerular filtration rate, *IMiD* immunomodulatory imide drug, *IQR* interquartile range), *ISS* international staging system, *PI* proteasome inhibitor, *VGPR* very good partial response.

*P*-values were computed using the Chi-square or Fisher’s exact test for comparisons of categorical variables and the *t* test for continuous variables.

Compared with Whites, Blacks were less likely to receive triplet therapies (55% vs. 73%, *P* < 0.001), including combined proteasome inhibitor (PI)/immunomodulatory imide drug (IMiD)-based (35% vs. 46%) or alkylator-based triplet therapy (20% vs. 27%). In addition, Blacks were significantly less likely than Whites to receive firstline autologous stem cell transplant (39% vs. 49%, *P* = 0.04) and triplet induction combined with firstline ASCT (33% vs. 44%, *P* = 0.04). Of those who received ASCT, there was no racial difference in receiving post-ASCT maintenance therapy. Interestingly, compared with Whites, Blacks with no or one HRCA were less likely to receive ASCT (37% vs. 50%, *P* = 0.02) or triplet therapy and ASCT (31% vs. 44%, *P* = 0.01), but they were more likely to receive ASCT or triplet therapy and ASCT when they had ≥2 HRCA (data not shown).

**Fig. 1:**
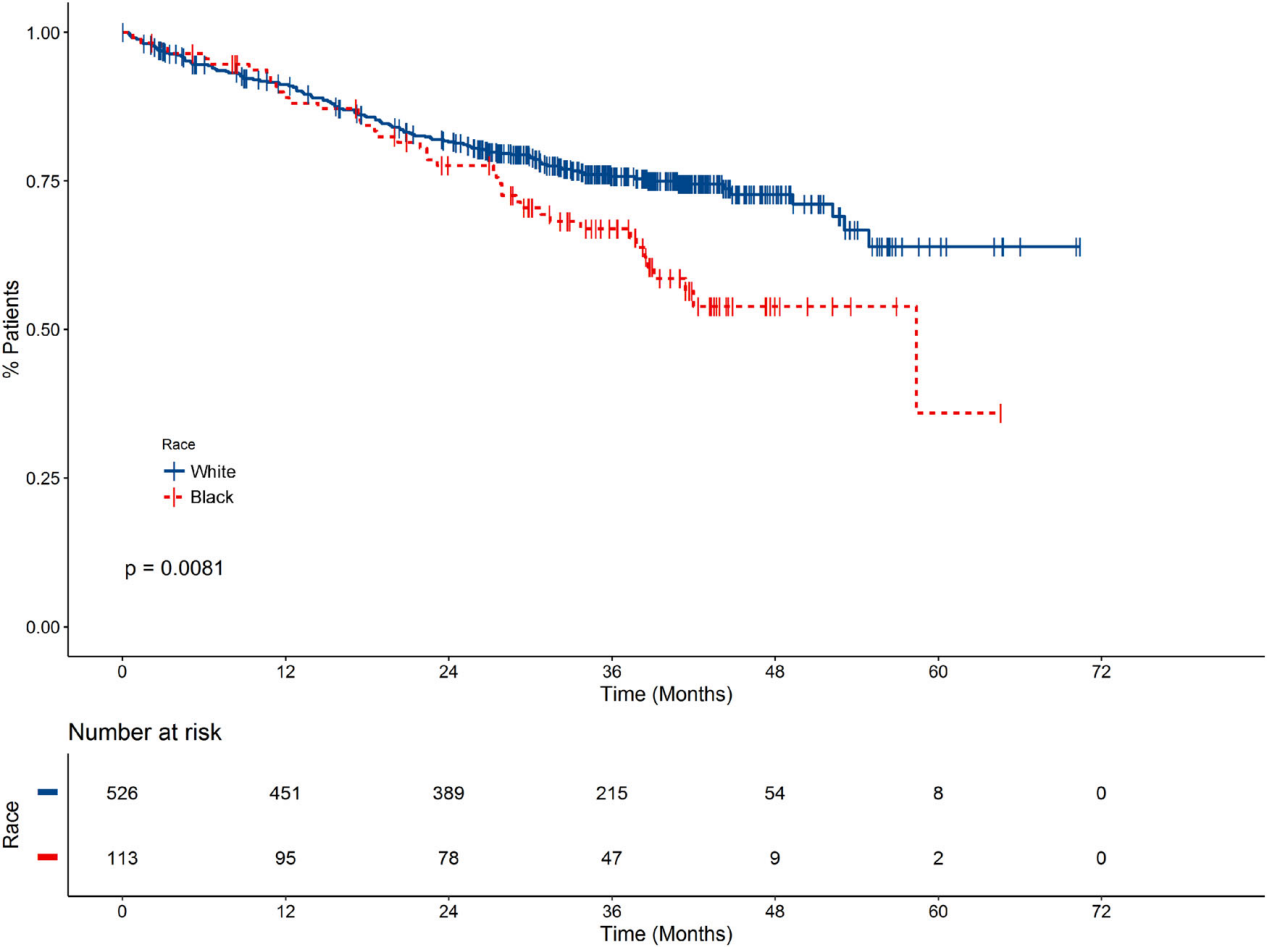
Overall survival stratified By race. Overall survival was shorter for Blacks compared with Whites, with an age-adjusted hazard ratio of 1.7 (95% confidence interval 1.2–2.4, *p* = 0.003).

**Fig. 2:**
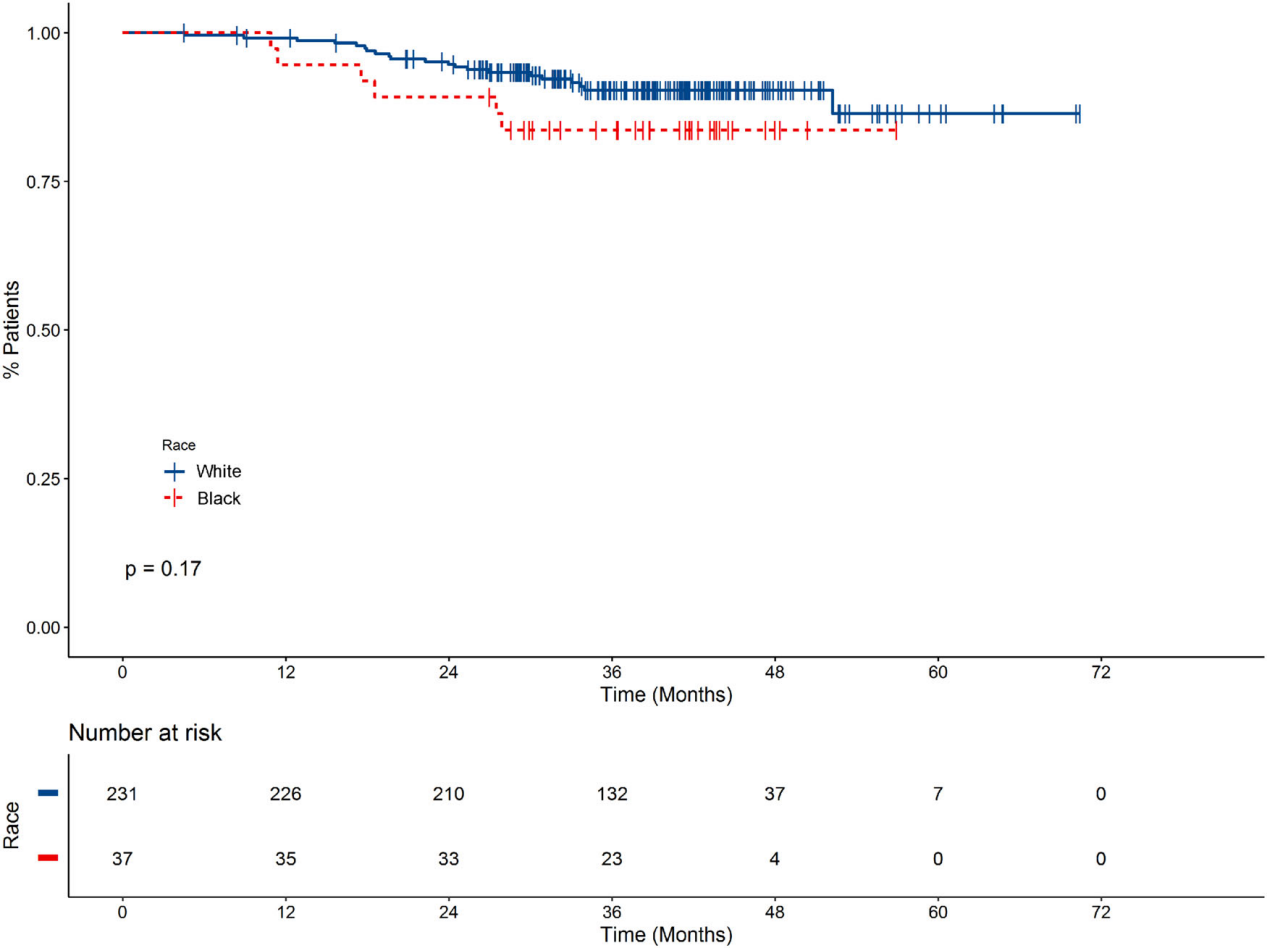
Overall survival of patients receiving triplet therapy and autologous transplant stratified by race. The difference in OS between races was partly attenuated in patients receiving triplet therapy and autologous stem cell transplant.

In both Blacks and Whites, age ≥65 was associated with both inferior PFS and OS (Supplementary Table 1). Age-adjusted univariate analysis in the whole cohort showed that both inferior PFS and OS were associated with male gender, Black race, ECOG PS ≥ 2, increasing ISS, eGFR ≤60, presence of ≥1 HRCA (OS only), presence of ≥2 HRCA, high risk by UAMS70, no triplet induction, and no ASCT (Supplementary Table 1). As shown in Fig. 1, OS was shorter for Blacks compared with Whites (age-adjusted hazard ratio (HR) 1.7, 95% confidence interval (CI) 1.2–2.4, *P* = 0.003). However, the difference in OS was attenuated in patients receiving triplet therapy and autologous transplant (Fig. 2). Multivariate analysis showed that increase in ISS, increase in number of HRCA, high risk by UAMS70, and no ASCT remained significantly associated with worse OS and PFS in Whites; male gender was also associated with inferior OS in Whites (Table 2). However, in Blacks, only the lack of frontline ASCT was associated with worse PFS and OS.

**Table 2:** Multivariate analysis of MMRF cohort.

Variable	PFS HR (95% CI, *P*)			OS HR (95% CI, *P*)		
	MMRF cohort (*n* = 639)	White (*n* = 526)	Black (*n* = 113)	MMRF cohort (*n* = 639)	White (*n* = 526)	Black (*n* = 113)
Age ≥65	1.2 (0.9–1.6, *P* = 0.2)	1.2 (0.9–1.7, *P* = 0.2)	0.8 (0.4–1.6, *P* = 0.6)	1.3 (0.9–2.0, *P* = 0.2)	1.2 (0.7–1.9, *P* = 0.5)	1.8 (0.8–4.2, *P* = 0.2)
Male gender	1.1 (0.9–1.5, *P* = 0.4)	–	1.4 (0.7–2.8, *P* = 0.3)	1.5 (0.97–2.2, *P* = 0.07)	1.6 (1.0–2.6, *P* = 0.047)	–
Black race	1.2 (0.8–1.7, *P* = 0.3)	N/A	N/A	1.4 (0.9–2.3, *P* = 0.1)	N/A	N/A
ECOG PS ≥ 2	0.97 (0.7–1.4, *P* = 0.9)	0.9 (0.6–1.3, *P* = 0.6)	–	1.0 (0.7–1.6, *P* = 0.9)	0.9 (0.5–1.5, *P* = 0.7)	–
Increase in ISS	1.3 (1.04–1.5, *P* = 0.02)	1.3 (1.0–1.6, *P* = 0.03)	1.4 (0.9–2.1, *P* = 0.2)	1.5 (1.1–2.0, *P* = 0.008)	1.8 (1.3–2.5, *P* = 0.001)	0.94 (0.5–1.8, *P* = 0.9)
eGFR ≤60	0.95 (0.7–1.3, *P* = 0.8)	1.0 (0.7–1.4, *P* = 0.98)	–	1.1 (0.7–1.7, *P* = 0.6)	1.1 (0.7–1.8, *P* = 0.8)	1.2 (0.5–3.0, *P* = 0.6)
Increase in number of HRCA	1.3 (1.1–1.5, *P* = 0.007)	1.3 (1.1–1.6, *P* = 0.01)	1.1 (0.7–1.7, *P* = 0.6)	1.5 (1.2–1.9, *P* = 0.001)	1.5 (1.2–2.0, *P* = 0.002)	1.1 (0.7–1.9, *P* = 0.7)
High risk by UAMS70	1.6 (1.2–2.4, *P* = 0.006)	1.6 (1.1–2.4, *P* = 0.03)	1.6 (0.7–3.9, *P* = 0.2)	1.9 (1.2–3.0, *P* = 0.004)	2.0 (1.2–3.4, *P* = 0.01)	2.1 (0.7–6.2, *P* = 0.2)
Non-triplet induction	0.8 (0.6–1.1, *P* = 0.2)	0.9 (0.6–1.2, *P* = 0.5)	–	1.0 (0.7–1.5, *P* = 0.9)	1.1 (0.7–1.8, *P* = 0.6)	–
No ASCT	2.5 (1.9–3.5, *P* < 0.001)	2.4 (1.7–3.3, *P* < 0.001)	2.5 (1.2–4.9, *P* = 0.01)	3.1 (1.9–5.0, *P* < 0.001)	3.5 (2.0–6.2, *P* < 0.001)	2.9 (1.2–7.1, *P* = 0.02)

*ASCT* autologous stem cell transplant, *ECOG PS* Eastern Cooperative Oncology Group performance status, *HRCA* high-risk cytogenetic abnormality [*t*(4;14), *t*(14;16), *t*(14;20), 1q gain, deletion 17p], *ISS* international staging system, *MMRF* Multiple Myeloma Research Foundation, *N/A* not applicable, *UAMS70* 70-gene expression profile.

“—” Indicates variables that were not included in the multivariate analysis.

*P*-values were computed using Cox proportional hazard models.

Given the persistent effect of triplet induction therapy and ASCT (triplet + ASCT) on OS, we performed univariate and multivariate analysis on this subgroup of patients. The effect of black race on OS appears to have only been partly mitigated by the receipt of triplet + ASCT (age-adjusted HR 2.3, 95% CI 0.9–5.8, *P* = 0.08) (Supplementary Table 1). When controlling for age (categorical), gender, ECOG PS, ISS, eGFR, and receipt of triplet + ASCT, Cox modeling shows again that the presence of 1 or 2+ HRCA had an impact on OS for Whites, but not for Blacks (Table 3).

**Table 3:** Racial difference in PFS and OS.

Variable	PFS		OS	
	White	Black	White	Black
	HR (95% CI)			
*HRCA*^a^				
0	1 (Reference)	1 (Reference)	1 (Reference)	1 (Reference)
1	1.3 (0.95–1.7)	1.1 (0.6–2.0)	1.9 (1.2–3.0)	0.8 (0.4–1.7)
2+	2.2 (1.5–3.2)	1.5 (0.7–3.3)	3.9 (2.3–6.6)	1.4 (0.5–3.7)
*ASCT*^b^				
No	1 (Reference)	1 (Reference)	1 (Reference)	1 (Reference)
Yes	0.4 (0.3–0.6)	0.2 (0.1–0.4)	0.3 (0.2–0.5)	0.5 (0.2–1.3)
*Frontline therapy*^b^				
No ASCT	1 (Reference)	1 (Reference)	1 (Reference)	1 (Reference)
ASCT, no triplet	0.7 (0.4–1.3)	0.1 (0.02–0.5)	0.7 (0.3–1.8)	0.8 (0.2–3.6)
ASCT + any triplet	0.4 (0.3–0.5)	0.2 (0.1–0.5)	0.3 (0.2–0.5)	0.4 (0.1–1.3)

*ASCT* autologous stem cell transplant, *HRCA* high-risk cytogenetic abnormality, *triplet* combination therapy involving three drugs including corticosteroids, a proteasome inhibitor, and either an alkylator or immunomodulatory imide drug.

^a^Model adjusted for age (categorical age), sex, ECOG PS, ISS, eGFR (≤60 vs. >60), and ASCT + triplet.

^b^Model adjusted for age (categorical age), sex, ECOG PS, ISS, eGFR (≤60 vs. >60), and HRCA.

## Discussion

In this large longitudinal cohort of newly diagnosed MM patients receiving modern treatment approaches, we show that Blacks had inferior OS compared with Whites and that this risk was only partly abrogated by receipt of triplet therapy and ASCT. Our findings of worse OS in Blacks than Whites are not consistent with previous studies. Using the SEER registries from 1973 to 2005, Waxman et al. found that Blacks experienced superior disease-specific survival and OS compared with Whites with MM^2^. This superior relative survival for Blacks was confirmed when expanding to the SEER registries from 1974 to 2014^7^. However, one focused analysis of SEER data from 2007 to 2011 found no difference in OS^8^, and another from 2007 to 2013 found that Blacks had superior MM-specific survival but not OS compared with Whites^9^. A single-center analysis of 170 Blacks and age- and gender-matched Whites with MM between 2002 and 2008 found no difference in overall survival (OS) at 35 month follow-up^10^. Ailawadhi et al. examined outcomes of Blacks and non-Blacks from pooled data of nine large cooperative group clinical trials conducted between 1988 and 2011 and also found no difference in survival^11^. These studies have for the large part included eras where state-of-the-art therapy approaches such as PIs and IMiDs were nonexistent or underutilized. The largest study to date—a VA study conducted by Fillmore et al.^13^—showed superior OS for Blacks compared with Whites with ~1400 patients having received a PI and IMiD as frontline therapy, but this is not directly comparable to our study because: (1) the percentage of patients who received novel induction regimens was much lower in the VA study, (2) ~98% of patients in the VA study were males, which we show to be an adverse prognostic factor, and (3) there was a lack of clinical annotation with cytogenetic data. In addition, the VA study found that the OS benefit for Black race was limited to those <65 years old at MM diagnosis (no racial difference in OS for those ≥65 years old). Indeed, nearly all population-based studies or those using administrative data (e.g., SEER-Medicare-linked data) lack prognostic information such as disease severity or cytogenetic risk stratification that could have contributed to treatment outcomes. It is also important to note that the patients included in our analysis have had access to improved therapeutic modalities for later lines of treatment (including monoclonal antibodies elotuzumab and daratumumab) compared with older cohorts.

In the current report, we found that the frequency of the number and type of HRCA were similar between races, which has been confirmed in an analysis of the Cancer Outcomes Tracking and Analysis (COTA) real-world database as well^22^. In contrast, a prior study found that Blacks were less likely to harbor *t*(11;14), *t*(4:14), monosomy 13, and monosomy 17 determined by fluorescent in situ hybridization (FISH)^19^. However, that analysis was limited by the restriction to only four cytogenetic abnormalities, the heterogeneity of FISH probes, and the lack of uniform CD138^+^ selection for FISH analysis which likely led to false negatives and underreported cytogenetic abnormalities. This current report circumvented these issues as cytogenetic abnormalities were inferred from NGS.

Increasing numbers of HRCA has been associated with inferior outcomes^23^, giving rise to terminology such as “single-hit” to describe the presence of one HRCA and “double-hit” when two HRCAs are present. While the presence of HRCA (single-hit or double-hit) in our study had a significant impact on survival in White patients, this was not the case for Black patients even after accounting for access to optimal frontline therapy. This discrepancy is likely not accounted for by superior responses among Black patients with HRCA, as the ≥VGPR rate was 22% for Black patients compared with 49% for White patients with HRCA. Alternatively, this may be due partly to our finding of differences in receiving ASCT or triplet therapy across different HRCA group between Blacks and Whites. We found that Blacks with 0 or 1 HRCA were less likely to receive ASCT or ASCT + triplet, whereas Blacks with 2+ HRCA were more likely to receive ASCT and ASCT + triplet, compared with Whites. This disparity may be attributed to implicit bias among physicians against ASCT in Blacks and requires further investigation. This may also reflect the fact that prior studies of cytogenetics in MM have used pooled data from clinical trials, which comprise patients predominantly of Caucasian backgrounds^24^. This study also suggests that high-risk gene expression profile by UAMS70 may be associated with PFS and OS in Blacks, though the confidence interval was wide. Overall, our findings show that whereas conventional HRCA has been used to determine the intensity of frontline therapy, this needs to be separately considered and tailored for black patients. Gene expression profiling may also be an important prognostic tool for Black patients, but this requires further validation in a larger cohort.

We found that many baseline MM characteristics were similar between Blacks and Whites, including the presence of renal dysfunction. Renal dysfunction at diagnosis for MM may be associated with lower relative OS—in particular when renal recovery does not occur with treatment—but prior data also suggest that Blacks may experience greater renal recovery than Whites^25–27^. Our findings of no difference between Black and White individuals on several clinical features are not entirely consistent with previous reports^2,11^. Given that patients included in this analysis were part of a prospective data collection research effort, it is possible that characteristics and treatments received by these patients are more representative of the centers that participated in the CoMMpass study rather than the entire MM patients population at large. Importantly, the proportion of Black patients in this study (18%) reflects the proportion of newly diagnosed Black MM patients (18–24%) in the United States^9,28^.

Though the use of frontline triplet induction therapy with a PI and IMiD was higher in Whites than Blacks (46% vs. 35%, *P* = 0.05), these rates are much higher than previously reported, such as in a study of VA patients (12.7% Whites vs. 8.8% Blacks, *P* < 0.001)^13^. Similarly, the rate of frontline transplant utilization was higher in Whites than Blacks (49% vs. 39%, *P* = 0.05), but this also exceeds previously reported data (as low as 9.7% in Whites and 9.3% in Blacks, and as high as 37.8% in Whites and 20.5% Blacks)^9,15^. This suggests that our study population represents a modern real-world one that is enriched for patients who received standard-of-care frontline therapy, including triplet induction and ASCT.

This study has several strengths including the use of the MMRF data that were prospectively collected with highly annotated clinical indices to allow for in-depth analysis of clinical outcomes. Moreover, there were a substantial number of black patients included in the study. The study’s limitations include the lack of cytogenetic information for all participants in the whole MMRF registry, and that cytogenetic abnormalities were inferred from NGS in the CoMMpass database. However, this method was standardized across all patients and prior studies have shown that using NGS in this manner achieves accuracy comparable to FISH^29,30^.

We have shown that Blacks had inferior OS compared with Whites, and this effect was not completely abrogated by controlling for access to standard-of-care regimens such as triplet induction and ASCT. That these surrogates of socioeconomic status do not explain the differences in OS suggests there may be a yet undescribed interplay of socioeconomic or biologic underpinnings to racial disparities in MM. Attributing racial differences to biology must be approached with care, as socioeconomic differences can be mistaken for biologic ones^31^. Deep response rates were lower among Black patients, regardless of HRCA status; however HRCA did not track with survival outcomes in Blacks, underscoring that the lack of race-specific risk prognostication schema for Blacks may be a key limitation toward achieving equal access to tailored therapy. Further investigation of racial differences in gene expression, including changes at the epigenetic level, serve as promising leads to identify potential reasons for these disparities. This serves once again as a clarion call to narrow the barriers toward ensuring black patients have access to and are offered optimal MM therapy.

## Supplementary information

Supplemental Table 1
