# Supplementary material for: Racial differences in treatment and outcomes in multiple myeloma: a multiple myeloma research foundation analysis
Source: Blood Cancer J. 2020 Aug 7;10(8):80. doi: 10.1038/s41408-020-00347-6 (PMC7414120; doi:10.1038/s41408-020-00347-6)
Supplement: Supplementary file 1 — Supplemental Table 1 [file 41408_2020_347_MOESM1_ESM.docx]

**Supplemental Table 1:** Age-Adjusted Univariate Analysis of MMRF Cohort

|  | **MMRF Cohort**  **(n=639)** | **White**  **(n=526)** | **Black**  **(n=113)** | **Triplet + ASCT Subset (n=268)** |
| --- | --- | --- | --- | --- |
| **PFS**  Age > 65  Male gender  Black race  ECOG PS >2  Increase in ISS  eGFR <60  > 1 HRCA  > 2 HRCA  High Risk by UAMS70  Non-Triplet Induction  No ASCT | **HR (95% CI, *p*)**  1.7 (1.4-2.1, p<0.001)  1.3 (1.04-1.6, p=0.02)  1.4 (1.1-1.8, p=0.02)  1.4 (1.1-1.9, p=0.01)  1.4 (1.3-1.7, p<0.001)  1.4 (1.1-1.7, p=0.006)  1.2 (0.97-1.5, p=0.09)  1.7 (1.3-2.2, p<0.001)  2.1 (1.6-2.9, p<0.001)  1.4 (1.1-1.8, p<0.001)  3.0 (2.4-3.9, p<0.001) | **HR (95% CI, *p*)**  1.7 (1.4-2.2, p<0.001)  1.2 (0.97-1.6, p=0.09)  N/A  1.4 (1.0-1.9, p=0.04)  1.4 (1.2-1.6, p<0.001)  1.3 (1.04-1.7, p=0.02)  1.3 (1.0-1.6, p=0.049)  1.9 (1.4-2.5, p<0.001)  2.2 (1.5-3.0, p<0.001)  1.4 (1.1-1.8, p=0.01)  2.8 (2.1-3.6, p<0.001) | **HR (95% CI, *p*)**  1.7 (1.04-2.6, p=0.03)  1.7 (1.0-2.8, p=0.046)  N/A  1.6 (0.9-2.8 p=0.1)  1.7 (1.2-2.4, p=0.003)  1.4 (0.8-2.2, p=0.2)  0.97 (0.6-1.5, p=0.9)  1.0 (0.5-2.1, p=1)  2.4 (1.1-5.2, p=0.03)  1.3 (0.8-2.2, p=0.2)  4.6 (2.5-8.4, p<0.001) | **HR (95% CI, *p*)**  1.1 (0.7-1.7, p=0.6)  1.2 (0.8-1.8, p=0.4)  1.2 (0.7-2.1, p=0.5)  1.0 (0.5-2.0, p=0.98)  1.3 (1.0-1.6, p=0.07)  1.0 (0.6-1.6, p=0.97)  1.2 (0.9-1.5, p=0.3)  1.6 (0.9-2.7, p=0.1)  2.2 (1.2-4.0, p=0.01)  N/A  N/A |
| **OS**  Age > 65  Male gender  Black race  ECOG PS >2  Higher ISS  eGFR <60  > 1 HRCA  >2 HRCA  High Risk by UAMS70  Non-Triplet Induction  No ASCT | **HR (95% CI, *p*)**  2.2 (1.6-3.0, p<0.001)  1.8 (1.3-2.5, p=0.001)  1.7 (1.2-2.4, p=0.003)  2.0 (1.4-2.8, p<0.001)  2.1 (1.7-2.5, p<0.001)  1.9 (1.4-2.6, p<0.001)  1.6 (1.1-2.1, p=0.005)  2.3 (1.6-3.3, p<0.001)  3.2 (2.2-4.8, p<0.001)  1.9 (1.4-2.6, p<0.001)  4.3 (2.9-6.4, p<0.001) | **HR (95% CI, *p*)**  2.1 (1.4-3.0, p<0.001)  1.8 (1.2-2.7, p=0.003)  N/A  1.8 (1.2-2.8, p=0.005)  2.1 (1.6-2.6, p<0.001)  1.8 (1.2-2.5, p=0.003)  1.8 (1.3-2.7, p=0.001)  2.7 (1.8-4.0, p<0.001)  3.7 (2.4-5.6, p<0.001)  1.9 (1.3-2.7, p=0.001)  4.6 (2.9-7.3, p<0.001) | **HR (95% CI, *p*)**  2.7 (1.5-5.1, p=0.001)  1.5 (0.7-3.0, p=0.3)  N/A  2.3 (1.1-4.5, p=0.02)  1.8 (1.2-2.9, p=0.007)  2.1 (1.1-3.9, p=0.03)  0.9 (0.5-1.6, p=0.7)  1.3 (0.6-3.0, p=0.5)  2.4 (0.98-6.5, p=0.07)  1.3 (0.7-2.8, p=0.4)  2.8 (1.2-6.4, p=0.02) | **HR (95% CI, *p*)**  2.7 (1.3-5.8, p=0.01)  1.6 (0.7-3.6, p=0.2)  2.3 (0.9-5.8, p=0.08)  1.1 (0.2-4.6, p=0.9)  1.6 (0.98-2.6, p=0.06)  1.1 (0.5-2.5, p=0.8)  1.7 (1.1-2.7, p=0.03)  3.5 (1.4-8.9, p=0.009)  3.9 (1.5-10.0, p=0.004)  N/A  N/A |

**Abbreviations:** ASCT = autologous stem cell transplant; ECOG PS= Eastern Cooperative Oncology Group performance status; eGFR = estimated glomerular filtration rate (as measured by CKD-EPI); HRCA = high-risk cytogenetic abnormality [t(4;14), t(14;16), t(14;20), 1q gain, deletion 17p]; ISS = International staging system; MMRF = Multiple Myeloma Research Foundation; N/A = Not applicable; UAMS70 = 70-gene expression profile.
